# Exploring cognitive characteristics and impairments in bipolar disorder: Insights from the BiDiLoS-Ng pilot study

**DOI:** 10.1017/gmh.2024.125

**Published:** 2024-12-06

**Authors:** Frances Nkechi Adiukwu, Olufisayo Adesokun, Chinwendu Maryam Amuta-Igwe, Izuchukwu Metu, Isoboye Charles Jack

**Affiliations:** 1 Department of Mental Health, College of Health Sciences, University of Port Harcourt, Port Harcourt, Rivers State, Nigeria; 2 Department of Neuropsychiatry, University of Port Harcourt Teaching Hospital, Port Harcourt, Rivers State, Nigeria

**Keywords:** bipolar disorder, executive function, working memory, cognitive function, euthymia

## Abstract

Bipolar disorder (BD) is a leading cause of disability and is linked to cognitive and functional impairment, increased mortality from cardiometabolic disorders and bipolar disorder suicide. Few studies in sub-Saharan Africa have explored cognitive dysfunction in bipolar disorder. Our study explores the cognitive characteristics in a bipolar patient cohort in Nigeria and assesses its association with clinical and demographic variables.

40 participants from the Bipolar Disorder Longitudinal Study, at baseline, were included in the pilot study of the BiDiLos-Ng. Using a cross-sectional design, cognitive function was assessed using the Screen for Cognitive Impairment in Psychiatry. Multiple linear regression models were used to explore associations between dependent and independent variables.

Cognitive impairment was present in 41% of the bipolar cohort, it was not associated with the frequency of mood episodes, and higher educational level was associated with higher verbal fluency test scores (*p* = 0.02). Being in employment (*p* = 0.03), younger age (*p =* 0.00), and lower YMRS score (*p =* 0.006) were associated with higher working memory test scores.

The presence of mania symptoms during the euthymic phase of BD was associated with cognitive impairment. Executive function and working memory were linked to better academic and occupational attainment.

## Impact Statement

- Bipolar Disorder has remained a leading cause of disability causing significant impairment in major areas of functioning.
- Cognitive impairment is seen in both the acute phase of a bipolar disorder episode and in remission.
- The presence of cognitive impairment especially in areas of executive functioning and working memory has a negative impact on academic and occupational functioning.
- In view of the prevalence of cognitive deficit even in the euthymic state, clinical inquiry into cognitive functioning should be part of the routine assessment of bipolar disorder patients on follow-up care, possibly using a sensitive, brief, self-rating screening instrument.

## Introduction

Bipolar disorder (BD) is a severe mental disorder (Flaaten et al., 2024) characterised by periods of recurrent episodes of elated mood (mania or hypomania) and depressed mood, spaced by periods of relative mood stability (euthymia) (Xu et al., 2020; Flaaten et al., 2024) and impairment in many areas of functioning (Miskowiak et al., 2022a). BD has lifetime prevalence rates ranging from 0.8% to 2% for BD type 1 and type 2 (Pasovic et al., 2022), is associated with premature mortality, and is a main cause of disability accounting for about 7% of the DALYs caused by mental and substance use disorders as at 2010 (Esan and Esan, 2016; McIntyre et al., 2020). BD is a leading cause of disability especially in the young as it has been linked to cognitive and functional impairment and increased mortality from cardiometabolic disorders and BD suicide (Vieta et al., 2018; Gergel et al., 2024).

Previously the prognosis for BD was considered quite favourable; however, recent findings have suggested that disability and poor outcomes are common, even with adequate treatment (Esan and Esan, 2016). While treatment for BD typically targets clinical improvement and the acute syndromes resolve in most cases, the reality is that residual symptoms often persist during the euthymic period. These symptoms, which vary in severity, along with impaired functional and social outcomes, are more the norm rather than the exception (Mezes et al., 2022).

An important and often overlooked cause of functional and psychosocial impairment in BD is cognitive impairment. While it is now widely recognised that a substantial proportion of individuals with BD experience cognitive impairments in the acute phase of the illness, it is often assumed that the euthymic phase is free of cognitive impairment (Buoli et al., 2014). Recent studies have shown that cognitive deficits persist beyond the resolution of acute mood symptoms, and this contributes to the functional disability associated with BD (Cullen et al., 2016; Van Rheenen et al., 2020a).

It is well-documented that certain cognitive domains are particularly impacted in BD, notably attention, processing speed, memory and executive functioning (Flaaten et al., 2024). Cognitive impairment has been increasingly recognised as an integral part of the BD phenotype (Pasovic et al., 2022). Several studies have shown that both young and older adults with BD have significant cognitive impairment (Flaaten et al., 2024; Pasovic et al., 2022), especially with executive function and attention processing, compared to age-matched control participants (Pasovic et al., 2022). Residual depressive symptoms, poor clinical course and a higher number of previous manic episodes may have a negative effect on cognitive performance. At the same time, the impact of medications, especially lithium, on cognition varies and may depend on the patient’s clinical response (Keramatian et al., 2021).

Critically, we need a better understanding of the factors associated with cognitive impairments to advance the development of intervention strategies that directly target the remediation of cognitive dysfunction and/or prevent subsequent cognitive decline in the illness. As such data on cognitive function in BD cohorts from countries like Nigeria is limited. This is important considering the differences in sociocultural context that impact the assessment of cognition, the concept of recovery and the stigma that surrounds BD. Few studies in sub-Saharan Africa have explored the cognitive function of BD patients, with very small sample sizes and other methodological issues limiting the overall confidence in the reported findings (Miskowiak et al., 2022a). To address some of these issues, this study aims to take a preliminary look at the cognitive characteristics of our BD cohort and examine the relationships between demographic and clinical variables and cognitive scores among patients with BD using data from the BiDiLoS-Ng pilot study.

## Methods

### Study participants

The BD Longitudinal Study-Nigeria (BiDiLos-Ng) is a multi-centre observational cohort study on BD in Nigeria (Adiukwu et al., 2023). The BiDiLoS-Ng is a multicentre open longitudinal cohort study of BD in Nigerian patients. The aim is to study the socio-demographic, clinical, cultural and biological factors associated with BD in a longitudinal cohort (Adiukwu et al., 2023). This paper pertains to the patients seen at the primary site of the registry.

The primary site of the study is the University of Port Harcourt Teaching Hospital (UPTH). This analysis in this report is part of the pilot phase of BiDiLos-Ng which occurred between January 2023 and June 2023. Study participants from the BD registry established by the BiDiLos-Ng were called up and had their cognitive and other assessments done. Forty participants with a clinical diagnosis of BD were recruited into the pilot study.

## Study procedure

### Participant recruitment

The study protocol received ethical approval from the Hospital Research and Ethical Committee (HREC) UPTH. Each potential study participant in the bipolar registry was reached *via* telephone and invited to take part in the pilot study. Following the telephone conversation, participants found to be eligible to be included in the pilot phase (above 18 years of age and an assessment of clinical remission as at the last outpatient clinic visit) were asked to come in-person for inclusion into the pilot study. Participants were given detailed information on the nature of the pilot study, and the possibility of being enrolled in the longitudinal phase of the study, and written informed consent was obtained.

### Interview process

Demographic (age, sex, educational, and employment status) and clinical characteristics (previous number of mood episodes and current medication) as seen in the bipolar registry were confirmed by each participant during the in-person interview. A BD diagnosis was then confirmed by the clinician interviewer using the past manic and hypomanic episode section of Module 4a of the Structured Clinical Interview for DSM 5 (SCID 5) (‘APA – The Structured Clinical Interview for DSM-5®’ n.d.). The current mood states of the participants were then assessed using the Hamilton depression rating scale (HAMD) (Hamilton, 1986) for depressive symptoms and the Young Mania Rating Scale (YMRS) (‘Young Mania Rating Scale – an overview | ScienceDirect Topics’ n.d.) for mania symptoms. Cognitive function was assessed using the Screen for Cognitive Impairment in Psychiatry (SCIP) (Purdon, 2005a) form 1.

The SCIP was designed as a cognitive screening tool for affective and psychotic disorders and takes about 15–20 min to complete. It assesses global cognition (SCIP total score) and has 5 subscales that assess verbal learning (VLT), working memory (WMT), verbal fluency (VFT), delayed recall (VLTD), and psychomotor speed (PST) (Purdon, 2005a). A cutoff score of greater than or equal to 75 on the SCIP total score indicates cognitive functioning within normal limits, while scores less than 75, 65, and 55 indicate mild, moderate, and severe cognitive limitations respectively (Purdon, 2005b). The subscale scores are interpreted as standardised Z-scores with scores less than −1.67 indicating a limitation in that subscale (Purdon, 2005b). Z-scores are calculated using the formula:

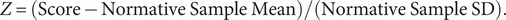



The normative sample was derived from n = 185 Canadians approximately 20 years of age with average to high average intellect (Purdon, 2005b). A Z-score of <−1.67 indicates a limitation in the cognitive subscale.

## Data analysis

Demographic and clinical variables were presented in tables. Mean +/− SD were used to summarise continuous variables and frequency and percentages were used for categorical variables. Global cognitive impairment (SCIP total sore) was grouped into present or absent using the cutoff score of 74 to determine the prevalence of cognitive impairment in the study participants. The severity of cognitive impairment was also determined using the relevant cutoff scores. To aid in the detailed interpretation of the subscale scores, standardised Z-scores were determined using the standard deviation of a normative sample (Purdon, 2005b) to determine the presence or absence of limitations in each subscale. Simple and multiple linear regression models were then used to determine the effect of clinical and demographic variables on cognitive function in the bipolar cohort. To achieve this, categorical variables were recoded as numerical variables (0,1,2 etc). Data was analysed using STATA 18. Dependent variables were tested for normality using the Shapiro-Wilks test of normality before regression analysis was done. The independent variables included in the multiple linear regression model were selected based on evidence from the literature (Larson et al., 2005; Biederman et al., 2011) and by running simple linear regression models on possible predictor variables to determine the potential for an association. Multicollinearity between variables was tested using variance inflation factor (VIF) and interaction terms and highly colinear covariates were removed from the regression model. To improve model fit, interaction terms (AGE*HAMD and AGE*YMRS) were included in the regression model, and lasso regression was carried out. This process was repeated using the subscale scores as the dependent variables.

## Results

Forty participants were enrolled on the study, but 1 participant dropped out of the study. Data was cleaned for analysis, with each missing value or observation deleted (this was done for educational level only). The mean age of the study participants was 34.42 ± 9.43 years. Most of the participants were male (58.97%) and had a tertiary level of education (89.57%). However, only 29% of the participants were in full-time employment (Table 1). The study participants were euthymic except for 2 participants who had HAMD scores above the cutoff for depression.

**Table 1.** Demographic and clinical variables of participants

Demographic variables	Mean	Std. dev.	Range
**Age**	34.42	9.43	19–54
**Sex**	**Freq.**	**Percent**	
Female	16	41.03	
Male	23	58.97	
Total	39	100	
**Education**			
Secondary	5	14.29	
Tertiary	30	85.71	
Total	35	100	
**Employment**			
Full-time	11	28.95	
Part-time	5	13.16	
Unemployed	22	57.89	
Total	38	100	
**Clinical variables**	**Mean**	**Std. dev.**	**Range**
Age at onset	27.26	10.99	13–54
Duration to diagnosis	15.81	40.33	0–54
Number of mania episodes	1.49	1.21	0–5
Number of depressed episodes	0.62	0.88	0–3
Number of hypomania episodes	0.08	0.35	0–2
Number of mixed episodes	0.38	0.54	0–2
Number of relapses	2.56	1.41	0–7
HAMD scores	1.56	2.69	0–11
YMRS scores	0.54	1.98	0–12

Cognitive impairment as measured using SCIP total score was normally distributed. Cognitive impairment was present in 16/39 (41%) of the bipolar cohort using a cutoff score of 74. Of the participants with cognitive impairment, half (50%) of them had mild impairment (Table 2). We observed that 43.6%, 46.2%, and 51.3% of participants had some limitation in delay recall, verbal learning and working memory respectively (Table 3).

**Table 2.** Prevalence of cognitive impairment in participants

Cognitive impairment (CI)	Freq.	Percent
Present	16	41.03
Absent	23	58.97
**Total**	**39**	**100**
**Cognitive impairment severity**		
Mild CI	8	50
Moderate CI	6	37.5
Severe CI	2	12.5
**Total**	**16**	**100**

**Table 3.** Subscale scores and prevalence of impairment

SCIP subscales	Mean	Std. dev.
Verbal learning test	19.03	3.61
Working memory test	16.59	5.20
Verbal fluency test	15.90	3.14
Verbal learning test delay	4.64	2.11
Processing speed test	23.29	6.43
SCIP Total	78.85	14.23
**Verbal learning test**	**Freq.**	**Percent**
No limitation	21	53.85
Some limitation	18	46.15
**Total**	**39**	**100**
**Working memory test**		
No limitation	19	48.72
Some limitation	20	51.28
**Total**	**39**	**100**
Verbal fluency test		
No limitation	39	100
**Total**	**39**	**100**
**Verbal learning test delay**		
No limitation	22	56.41
Some limitation	17	43.59
**Total**	**39**	**100**
**Processing speed test**		
No limitation	35	89.74
Some limitation	4	10.26
**Total**	**39**	**100**

The total number and type of mood episodes did not have a significant relationship with global cognitive score (SCIP total) (Table 4). To control for the effect of clinical and demographic variables, we included these variables in a linear regression model (Table 5a and 5b) and did not find any significant effect on the type and number of mood episodes on the SCIP total score. However, we found that about a 1-year decrease and 5.03 unit decrease in age and young mania scores respectively were associated with a 1 unit increase in SCIP score (*p* = 0.03, and *p* = 0.02 respectively) when controlling for the total number of relapses of the study participant. However, this model did not provide strong evidence that the predictors used significantly explain the variability in the dependent variable (Prob > F = 0.15). In a sensitivity analysis, we added interaction terms (to the multiple linear regression model (Table 5b) to explore possible interactions between variables. This did not improve the overall model fit (Prob > F: 0.23).

**Table 4.** Linear regression of total SCIP score and frequency of mood episodes

SCIP total Score	Coefficient	Std. err.	t	P	[95% conf. interval]	
Total number of mood episodes	−1.37	1.64	−0.83	0.41	−4.70	1.96
_cons	82.36	4.79	17.18	0.00	72.65	92.07
**SCIP total score**						
Number of mania episodes	−0.83	2.02	−0.41	0.68	−4.93	3.27
Number of depressive episodes	0.10	2.83	0.04	0.97	−5.66	5.86
Number of hypomania episodes	−12.17	6.85	−1.78	0.08	−26.10	1.75
Number of mixed episodes	−1.01	4.32	−0.23	0.82	−9.80	7.77
_cons	81.35	4.89	16.64	0.00	71.41	91.28

**Table 5a.** Multiple linear regression of total SCIP score and frequency of mood episodes

SCIP total score	Coefficient	Std. err.	t	P > t	[95% conf. interval]	
Number of relapses	−1.75	2.21	−0.79	0.44	−6.38	2.88
educational level	7.08	8.05	0.88	0.39	−9.77	23.94
Employment status	−3.76	3.00	−1.25	0.23	−10.03	2.51
Age^a^	−0.90	0.38	−2.40	**0.03**	−1.68	−0.11
Duration to BD diagnosis	−0.01	0.08	−0.13	0.90	−0.17	0.15
HAMD score	2.53	1.52	1.66	0.11	−0.65	5.71
YMRS score^a^	−5.03	1.91	−2.63	**0.02**	−9.03	−1.03
_cons	112.15	14.32	7.83	0.00	82.16	142.13
**SCIP total score**						
Number of mania episodes	−1.60	2.45	−0.65	0.52	−6.77	3.56
Number of depressive episodes	−5.98	4.46	−1.34	0.20	−15.39	3.43
Number of hypomania episodes^b^	0.00	(omitted)				
Number of mixed episodes	−0.33	5.33	−0.06	0.95	−11.58	10.92
educational level	2.95	9.17	0.32	0.75	−16.39	22.29
Employment status	−3.56	3.06	−1.16	0.26	−10.02	2.91
Age	−0.79	0.42	−1.90	0.08	−1.67	0.09
Duration to BD diagnosis	−0.02	0.08	−0.25	0.81	−0.20	0.16
HAMD score	2.61	1.67	1.56	0.14	−0.92	6.14
YMRS score^a^	−5.10	2.04	−2.50	**0.02**	−9.40	−0.79
_cons	113.58	15.01	7.57	0.00	81.90	145.25

a Statistical significance.

b omitted due to multicollinearity.

**Table 5b.** Multiple linear regression of total SCIP Score and frequency of mood episodes with variable interactions

SCIP total score	Coefficient	Std. err.	t	P > t	[95% conf. interval]	
Number of relapses	−1.79	2.32	−0.77	0.45	−6.66	3.08
educational level	7.06	8.28	0.85	0.41	−10.34	24.45
Employment status	−3.72	3.13	−1.19	0.25	−10.29	2.86
Age	−0.88	0.47	−1.85	0.08	−1.88	0.12
Duration to BD diagnosis	−0.01	0.08	−0.10	0.92	−0.18	0.17
HAMD Score	2.93	5.61	0.52	0.61	−8.87	14.72
YMRS score	−5.18	2.79	−1.86	0.08	−11.05	0.69
AGE_HAMD_interaction	−0.01	0.14	−0.07	0.94	−0.30	0.28
_cons	111.45	17.46	6.38	0.00	74.76	148.13
**SCIP total score**						
Number of mania episodes	−1.68	2.63	−0.64	0.53	−7.25	3.90
Number of depressive episodes	−6.02	4.62	−1.30	0.21	−15.81	3.76
Number of hypomania episodes^a^	0.00	(omitted)				
Number of mixed episodes	−0.25	5.55	−0.04	0.97	−12.02	11.53
educational level	2.94	9.45	0.31	0.76	−17.08	22.97
Employment status	−3.49	3.22	−1.08	0.30	−10.32	3.34
Age	−0.77	0.50	−1.53	0.15	−1.83	0.30
Duration to BD diagnosis	−0.02	0.09	−0.22	0.83	−0.21	0.17
HAMD score	3.19	5.80	0.55	0.59	−9.12	15.49
YMRS score	−5.30	2.86	−1.85	0.08	−11.36	0.77
AGE_HAMD_interaction	−0.02	0.15	−0.10	0.92	−0.33	0.29
_cons	112.64	17.92	6.28	0.00	74.64	150.64

a Omitted due to multicollinearity.

The effect of independent variables on the subscale scores was carried out (Table 6). Higher educational level was associated with higher verbal fluency test scores (Coeff. = 4.49, *p* = 0.02). Higher employment status (Coeff. = 2.09, *p* = 0.03), younger age (Coeff. = −0.384, *p =* 0.00), and lower YMRS score (Coeff. = −1.66, *p =* 0.006) were associated with higher working memory test scores. Higher employment level (Coeff. = 1.23, *p* = 0.03) and younger age (Coeff. = −0.24, *p* = 0.000) were associated with higher verbal fluency test scores. None of the included covariates had a significant effect on delayed verbal learning tests and processing speed test scores.

**Table 6.** Linear regression of SCIP subscale scores and clinical and demographic variables

Verbal learning test score	Coeff.	Std. err.	t	P	[95% conf. interval]	
Educational level^a^	4.49	1.72	2.60	**0.02**	0.95	8.02
Employment status	−0.17	0.74	0.23	0.82	−1.35	1.69
Age	−0.11	0.07	−1.53	0.14	−0.26	0.04
HAMD score	0.57	0.36	1.60	0.12	−0.16	1.30
YMRS score^a^	−0.94	0.46	−2.05	**0.05**	−1.88	0.00
_cons	18.36	3.21	5.72	0.00	11.77	24.95
**Working memory test score**						
Educational level	2.306	2.107	1.090	0.283	−2.017	6.628
Employment status^a^	2.093	0.905	−2.310	**0.029**	−3.950	−0.236
Age^a^	−0.384	0.089	−4.300	**0.000**	−0.568	−0.201
HAMD score	0.255	0.435	0.590	0.563	−0.638	1.148
YMRS score^a^	−1.664	0.561	−2.970	**0.006**	−2.815	−0.514
_cons	30.824	3.924	7.860	0.000	22.773	38.875
**Verbal fluency test score**						
Educational level	1.62	1.29	1.26	0.22	−1.02	4.26
Employment status^a^	1.23	0.55	−2.23	**0.03**	−2.36	−0.10
Age^a^	−0.24	0.05	−4.33	**0.00**	−0.35	−0.12
HAMD score	0.37	0.27	1.41	0.17	−0.17	0.92
YMRS score	−0.51	0.34	−1.50	0.15	−1.22	0.19
_cons	23.94	2.39	10.00	0.00	19.02	28.85
**Verbal learning delay test score**						
Educational level	2.10	1.05	2.00	0.06	−0.05	4.26
Employment status	0.19	0.45	−0.42	0.68	−1.11	0.74
Age	−0.07	0.04	−1.58	0.13	−0.16	0.02
HAMD score	0.11	0.22	0.50	0.62	−0.34	0.55
YMRS score	−0.42	0.28	−1.48	0.15	−0.99	0.16
_cons	5.33	1.96	2.72	0.01	1.31	9.35
**Processing speed test**						
Educational level	1.99	3.50	0.57	0.58	−5.20	9.17
Employment status	−0.37	1.50	0.25	0.81	−2.70	3.45
Age	−0.14	0.15	−0.95	0.35	−0.45	0.17
HAMD score	−0.05	0.72	−0.07	0.95	−1.53	1.43
YMRS score	−0.24	0.93	−0.25	0.80	−2.14	1.67
_cons	25.88	6.51	3.98	0.00	12.50	39.27

a Statistical significance.

## Discussion

### Cognitive impairment in the BiDiLos-Ng cohort

We assessed the cognitive function of patients with BD using the Screening for Cognitive Impairment in Psychiatry (SCIP) and found that 41% of our BD cohort had some level of cognitive impairment with varying degrees of severity. While our study did not have a control group to determine an association between BD and cognitive impairment, other studies have shown that this association exists. Cullen et al. (2016) in their systematic review of cognitive impairment in BD patients, found that cognitive impairment in BD was higher than comparison groups in almost all cognitive measures and at every cutoff (Cullen et al., 2016). There are significant variations within published literature on the prevalence of cognitive impairment in BD, majority of these studies align with our findings that a significant proportion of BD patients have varying degrees of cognitive impairments either globally or in specific domains (Keramatian et al., 2021). Cognitive impairment varied within the cohort when specific cognitive domains were assessed.

We found that the prevalence of impairment in executive function, working memory, and delayed recall was higher when compared to the prevalence derived from the total SCIP score. This implies that people living with BD who do not exhibit global cognitive decline might have limitations in specific domains of cognitive function which may impair areas of their functioning. There is consistent evidence of cognitive impairment in BD in areas of executive functioning, verbal working memory and sustained attention and these specific impairments have also been seen in first-degree relatives of people with BD (Bora et al., 2008).

## Mood episodes and frequency and cognitive impairment in BD

Using linear regression models, we investigated the effect of the frequency of relapses (total number of mood episodes) and frequency of specific mood episodes on cognitive impairment in our cohort. We found no significant relationship between the frequency of mood episodes and relapses in cognitive function (SCIP total score). The frequency of individual mood episodes and the total number of relapses showed a negative association with the total SCIP score, but this association was not statistically significant. However, the imprecision of our estimates suggests that we lacked the power to detect significant effects.

Some studies directly comparing single to multiple mood episodes have shown worse cognitive impairment in BD patients with multiple mood episodes indicating a progressive cognitive dysfunction (Van Rheenen et al., 2020b). However, this has not been supported in more recent prospective studies where long-term stability of cognitive function across all domains has been found, indicating no cognitive deterioration (Flaaten et al., 2024). Systematic reviews have also found no consistent evidence for an association between cognitive impairment and subsequent mood episodes (Miskowiak et al., 2022b). This implies that the cognitive impairment seen in people living with BD might be a core feature of the disorder and this may be amenable to cognitive therapy (Ott et al., 2020; Miskowiak et al., 2021).

## Factors associated with cognitive impairment in BD

We used multiple linear regression to determine variables associated with cognitive impairment in our cohort. We found that the presence of mania symptoms in the euthymic state is associated with lower scores on tests of executive function and working memory. The negative effect of mania symptoms on executive function has been consistently reported in the literature (Larson et al., 2005). Contrary to popular belief that the euthymic phase of BD (remission) is not associated with psychopathologies, studies are consistently showing that this is not the case. Recently, a study carried out in Nigeria showed that BD I in remission was not different from schizophrenia in remission when comparing the severity of their psychopathologies (Esan et al., 2017). The presence of mood variability during the euthymic phase of BD has negative effects on cognitive functioning as seen in our study, and as such may impair occupational and academic functioning and possibly quality of life.

Higher educational attainment was associated with better performance in tests of executive function and being gainfully employed (part- or full-time employment) was associated with better performance in tests of executive function and working memory. While we cannot conclude on the direction of these associations, there is evidence from literature that higher educational performance may be a distinctive feature of BD *i.e.* when comparing the educational attainment of patients with BD against other severe mental disorders, patients with BD tended to have higher educational achievement before their first episode (Vreeker et al., 2016). This was also seen in our study where most of our cohort had a tertiary level of education and having a higher level of education was associated with less cognitive impairment. Studies have also shown that deficits in executive dysfunction in patients with BD have been associated with lower academic achievements (Biederman et al., 2011) and that BD patients who are actively employed have better executive function (Drakopoulos et al., 2020). Cognitive performance in BD is a predictor of favourable employment status, with verbal memory and executive functioning predicting work functioning (Gilbert and Marwaha, 2013; Tse et al., 2014). Cognitive remediation therapy (CRT) has been shown to improve the quality of working life in people with severe mental illnesses (Ikebuchi et al., 2017) with people who undergo CRT having higher employment percentages than those who did not undergo CRT (Rodríguez Pulido et al., 2021). In BD a study has found that CRT increases overall occupational and psychosocial functioning (Deckersbach et al., 2010). We can therefore propose that better educational attainment is associated with better executive function, which in turn could be a powerful predictor of occupational status in BD. This, thus highlights the need to incorporate cognitive assessment as an integral part of BD follow-up care alongside the use of pharmacotherapy and psychotherapy to improve the overall outcome of the disorder.

## Limitation and recommendation

In our pilot study, all participants were recruited in the euthymic state and as such the full effect of mania and depressive symptoms on cognitive function was not studied. We did not include a control group in the pilot study, and we used a cross-sectional design to find associations. While we used a standardised population to determine the Z-score, this standard population does not reflect the peculiarities of our population. Our sample size of 39 is too small for this study to have sufficient power to detect significant associations and is less likely to accurately reflect the true characteristics of the sample population. This emphasises the need for a larger bipolar cohort in Nigeria. To this end, the longitudinal study intends to recruit more participants over 2 study sites, thus more data will be collected including a control group, the participants will then be followed up longitudinally to improve sample size and thus improve the power of the study.

## Strengths

Our study is one of the first from our population to study the cognitive function in BD patients considering the number of illness episodes, educational attainment, and sociocultural clime.

## Conclusions

Cognitive impairment (global and specific domains) is highly prevalent in patients living with BD. These impairments might be core features of BD affecting overall functioning, yet they tend to be overlooked in the management of the disorder. While there are controversies concerning the efficacy of treatments for cognitive impairments, our findings highlight the importance of more research in this field especially in sub-Saharan Africa.

## Supporting information

Adiukwu et al. supplementary material Adiukwu et al. supplementary material

## Data Availability

The study data are available from authors upon request.

## Supplementary material

The supplementary material for this article can be found at http://doi.org/10.1017/gmh.2024.125.
