# Supplementary material for: Exploring cognitive characteristics and impairments in bipolar disorder: Insights from the BiDiLoS-Ng pilot study
Source: Glob Ment Health (Camb). 2024 Dec 6;11:e120. doi: 10.1017/gmh.2024.125 (PMC11704381; doi:10.1017/gmh.2024.125)
Supplement: Adiukwu et al. supplementary material [file S2054425124001250sup001.docx]

Shapiro Wilks test for normality W = 0.969, *p* = 0.357

Figure 1. Normality test for total SCIP score (dependent variable)

**. Multiple Linear Regression of Total SCIP Score and Frequency of Mood Episodes with variable interactions**

| SCIP TOTAL SCORE | Coefficient | Std. err. | t | P>t | [95% conf. | interval] |
| --- | --- | --- | --- | --- | --- | --- |
| Number of relapses | -1.79 | 1.64 | -1.09 | 0.29 | -5.18 | 1.59 |
| Employment Status | -3.35 | 2.74 | -1.22 | 0.23 | -9.01 | 2.31 |
| AGE_YMRS_interaction | -0.75 | 0.32 | -2.36 | 0.03 | -1.40 | -0.09 |
| Duration to Diagnosis | 0.01 | 0.07 | 0.16 | 0.88 | -0.13 | 0.15 |
| HAMD Score | 3.09 | 1.76 | 1.75 | 0.09 | -0.55 | 6.73 |
| YMRS Score | -1.12 | 7.84 | -0.14 | 0.89 | -17.34 | 15.10 |
| AGE_YMRS_interaction | -0.20 | 0.41 | -0.48 | 0.64 | -1.04 | 0.65 |
| _cons | 112.34 | 13.91 | 8.07 | 0.00 | 83.56 | 141.12 |
|  |  |  |  |  |  |  |
| SCIP TOTAL SCORE |  |  |  |  |  |  |
| Number of Mania Episodes | -1.64 | 2.05 | -0.80 | 0.43 | -5.91 | 2.63 |
| Number of depression episodes | -6.56 | 3.64 | -1.80 | 0.09 | -14.16 | 1.04 |
| Number of Hypomania episodes | 9.98 | 18.78 | 0.53 | 0.60 | -29.19 | 49.15 |
| Number of Mixed Episodes | -1.21 | 4.63 | -0.26 | 0.80 | -10.86 | 8.44 |
| Employment Status | -3.56 | 2.89 | -1.23 | 0.23 | -9.59 | 2.48 |
| AGE_YMRS_interaction | -0.73 | 0.34 | -2.13 | 0.05 | -1.45 | -0.01 |
| Duration to Diagnosis | -0.01 | 0.07 | -0.17 | 0.86 | -0.17 | 0.14 |
| HAMD Score | 2.92 | 1.83 | 1.59 | 0.13 | -0.90 | 6.73 |
| YMRS Score | -3.74 | 10.62 | -0.35 | 0.73 | -25.89 | 18.42 |
| AGE_YMRS_interaction | -0.07 | 0.53 | -0.14 | 0.89 | -1.18 | 1.03 |

**Supplementary Table 2. Multiple Linear Regression of Total SCIP Score and Frequency of Mood Episodes with variable interactions**

| **SCIP TOTAL SCORE** | **Coefficient** | **Std. err.** | **t** | **P>t** | **[95% conf.** | **interval]** |
| --- | --- | --- | --- | --- | --- | --- |
| Number of Relapses | -1.79 | 2.32 | -0.77 | 0.45 | -6.66 | 3.08 |
| educational level | 7.06 | 8.28 | 0.85 | 0.41 | -10.34 | 24.45 |
| Employment Status | -3.72 | 3.13 | -1.19 | 0.25 | -10.29 | 2.86 |
| Age | -0.88 | 0.47 | -1.85 | 0.08 | -1.88 | 0.12 |
| Duration to BD diagnosis | -0.01 | 0.08 | -0.10 | 0.92 | -0.18 | 0.17 |
| HAMD Score | 2.93 | 5.61 | 0.52 | 0.61 | -8.87 | 14.72 |
| YMRS score | -5.18 | 2.79 | -1.86 | 0.08 | -11.05 | 0.69 |
| AGE_HAMD_interaction | -0.01 | 0.14 | -0.07 | 0.94 | -0.30 | 0.28 |
| _cons | 111.45 | 17.46 | 6.38 | 0.00 | 74.76 | 148.13 |
|  |  |  |  |  |  |  |
| **SCIP TOTAL SCORE** |  |  |  |  |  |  |
| Number of Mania Episodes | -1.68 | 2.63 | -0.64 | 0.53 | -7.25 | 3.90 |
| Number of Depressive Episodes | -6.02 | 4.62 | -1.30 | 0.21 | -15.81 | 3.76 |
| Number of Hypomania Episodes** | 0.00 | (omitted) |  |  |  |  |
| Number of Mixed Episodes | -0.25 | 5.55 | -0.04 | 0.97 | -12.02 | 11.53 |
| educational level | 2.94 | 9.45 | 0.31 | 0.76 | -17.08 | 22.97 |
| Employment Status | -3.49 | 3.22 | -1.08 | 0.30 | -10.32 | 3.34 |
| Age | -0.77 | 0.50 | -1.53 | 0.15 | -1.83 | 0.30 |
| Duration to BD diagnosis | -0.02 | 0.09 | -0.22 | 0.83 | -0.21 | 0.17 |
| HAMD Score | 3.19 | 5.80 | 0.55 | 0.59 | -9.12 | 15.49 |
| YMRS score | -5.30 | 2.86 | -1.85 | 0.08 | -11.36 | 0.77 |
| AGE_HAMD_interaction | -0.02 | 0.15 | -0.10 | 0.92 | -0.33 | 0.29 |
| _cons | 112.64 | 17.92 | 6.28 | 0.00 | 74.64 | 150.64 |

** omitted due to multicollinearity
