# Supplementary material for: Exploring cognitive characteristics and impairments in bipolar disorder: Insights from the BiDiLoS-Ng pilot study
Source: Glob Ment Health (Camb). 2024 Dec 6;11:e120. doi: 10.1017/gmh.2024.125 (PMC11704381; doi:10.1017/gmh.2024.125)
Supplement: Peer review history

# Editor report: Recommendation: Exploring cognitive characteristics and impairments in bipolar disorder: Insights from the BiDiLoS-Ng pilot study — R0/PR2

**DOI:** 10.1017/gmh.2024.125.pr2

**Affiliations:** University of Zimbabwe Faculty of Medicine, Zimbabwe

No accompanying comment.

# Editor report: Decision: Exploring cognitive characteristics and impairments in bipolar disorder: Insights from the BiDiLoS-Ng pilot study — R0/PR3

**DOI:** 10.1017/gmh.2024.125.pr3

**Affiliations:** London School of Hygiene & Tropical Medicine, United Kingdom of Great Britain and Northern Ireland

No accompanying comment.

# Editor report: Recommendation: Exploring cognitive characteristics and impairments in bipolar disorder: Insights from the BiDiLoS-Ng pilot study — R1/PR5

**DOI:** 10.1017/gmh.2024.125.pr5

**Affiliations:** University of Zimbabwe Faculty of Medicine, Zimbabwe

No accompanying comment.

# Editor report: Decision: Exploring cognitive characteristics and impairments in bipolar disorder: Insights from the BiDiLoS-Ng pilot study — R1/PR6

**DOI:** 10.1017/gmh.2024.125.pr6

**Affiliations:** London School of Hygiene & Tropical Medicine, United Kingdom of Great Britain and Northern Ireland

No accompanying comment.

# Editor report: Recommendation: Exploring cognitive characteristics and impairments in bipolar disorder: Insights from the BiDiLoS-Ng pilot study — R2/PR8

**DOI:** 10.1017/gmh.2024.125.pr8

**Affiliations:** University of Zimbabwe Faculty of Medicine, Zimbabwe

No accompanying comment.

# Editor report: Decision: Exploring cognitive characteristics and impairments in bipolar disorder: Insights from the BiDiLoS-Ng pilot study — R2/PR9

**DOI:** 10.1017/gmh.2024.125.pr9

**Affiliations:** London School of Hygiene & Tropical Medicine, United Kingdom of Great Britain and Northern Ireland

No accompanying comment.
